# Optimizing consumer choice in selection of health insurance

**DOI:** 10.1093/haschl/qxag191

**Published:** 2026-07-24

**Authors:** Miranda Yaver, David M Anderson, Risha Gidwani

**Affiliations:** Department of Health Policy and Management, University of Pittsburgh, Pittsburgh, PA 15222, United States; Department of Health Services Policy Management, University of South Carolina, Columbia, SC 29208, United States; Division of Health Care Policy and Research, University of Colorado School of Medicine, Aurora, CO 80045, United States; Department of Health Systems, Management and Policy, Colorado School of Public Health, Aurora, CO 80045, United States; RAND, Santa Monica, CA 90401, United States; Department of Health Policy and Management, UCLA Fielding School of Public Health, Los Angeles, CA 90095, United States

**Keywords:** health insurance literacy, access to care, health insurance inequity, administrative burden, consumer decision-making, health insurance, health policy

## Abstract

Patients are confronted with an overwhelming number of choices when enrolling in health insurance plans, with marketplace enrollees often having over 100 plan options and Medicare Advantage enrollees having on average 42 plan options. This can fuel confusion, administrative burden, and suboptimal plan selection, which in turn can be costly to the patient, who may face greater cost sharing and thus reduce even necessary health care utilization. This is particularly troubling because most Americans experience low levels of health insurance literacy as well as limited numeracy, making it especially challenging to adjudicate among plan options in this setting of health insurance complexity. Accurately discerning optimal plan choice is especially critical given America's notably high health care costs. While there are a growing number of navigator tools, including through the use of artificial intelligence, they tend to suffer from poor take-up and/or efficacy. Existing funding streams prioritize the assessment of the performance of existing interventions, but patients' challenges navigating health plan selection highlight the need to support research to both design and test new interventions to promote health insurance literacy and, in turn, access to necessary medical care.

Health insurance choice is a complex, opaque process with substantial financial consequences for inefficient or ineffective choice. Dominated choices, which occur when individuals choose a health insurance plan other than that which offers better benefits at the same or lower costs, are common.^1^ Choice errors are common across all types of insurance including employer-sponsored insurance, within and across Medicare programs, including prescription drug coverage, and the Affordable Care Act. These errors include failure to appropriately respond to price signals from both deductible and healthcare bills. This is especially problematic in an insurance landscape dominated by consumer-directed healthcare.

Consumer choice as a means of disciplining health care markets has not been comprehensively successful. Failures in consumer price shopping and service selection are well documented.^2^ These are systemic failures, which are generally most effectively and equitably resolved by federal policymaking to equitably reduce the probability of errors and the consequences of those errors. Federal solutions are more likely to be effective because self-insured employer-sponsored plans, covering roughly one-third of the American population, are exempt from state regulation. The most currently viable solutions, especially for proponents of consumer-directed markets, involve educating and empowering patients as consumers of health and health insurance.

In a notably complex and fragmented health insurance landscape, health insurance literacy (HIL) is especially critical, yet it is often limited among Americans. Health insurance literacy —or the ability to find, comprehend, select, and effectively utilize health insurance plans in order to obtain health care—requires literacy as well as numeracy.^3^ These skills are unevenly distributed, with racial, ethnic, language, and income disparities in comprehension of insurance terms, which can leave some patients behind, vulnerable to suboptimal plan selection and utilization. In the United States, 37% of nonelderly adults also report low levels of numeracy, with lowest numeracy levels found in White and/or native-born US populations.^4^ Thus, disparities in HIL do not fit the traditional pattern of disparities generally observed in healthcare. Low HIL is associated with higher patient financial burden and with delayed or foregone care.^5^

The scale of choice confronting patients underscores why HIL matters. Seniors must often navigate among 10 Medigap plans, 14 stand-alone prescription drug plans, and an average of 42 Medicare Advantage plans, sometimes while experiencing cognitive decline that complicates effective plan comparison. Affordable care act (ACA) marketplace enrollees face similarly daunting choice overload, confronted with over 100 plans on average.^6^ But more choice is not always better, as the quality of consumer decision-making declines with increased choice set.^7^

Plan selection is just one pressure point. Health insurance utilization presents its own unique set of problems, such as contending with delays and denials of coverage, which can prove quite challenging to understand and overcome. The average US adult reads at a fifth-grade level, while health insurance materials are typically written at a far more advanced level. The effects of low literacy could be mitigated by requiring that health insurance materials be written at no higher than a median reading level. Patients may also not fully grasp their rights or the steps required to secure coverage. Thus, health insurance complexity and low HIL interact to create substantial patient administrative burdens. Recent federal policy changes may further steer patients toward consumer-direct health plans, increasing patients' financial exposure and discouraging necessary medical care.

Low HIL thus has tangible consequences. It can lead to suboptimal plan selection, delays in care, unsuccessful efforts to overturn coverage denials, and consumer disempowerment. In a health care system characterized by notoriously high costs and high cost-sharing, these dynamics undermine access to care and intensify patients' financial strain.

Thus, in order to adequately support a consumer-directed market, consumers require sufficient HIL. As healthcare use is complex and depends on multiple, often time-varying, factors such as an individual's clinical profile, risk tolerance, insurance benefit design, level of disposable income, and realized access to appropriate providers, improving consumers' use of their health insurance requires multifaceted HIL solutions. Artificial intelligence (AI) tools to help consumers select the right insurance plan represent one promising solution, although it is unclear whether they are being built to adequately address use of health insurance and its funding (eg, health savings accounts). There is limited evidence on the use of AI and machine learning tools, although initial evidence suggests fewer dominated choices, less costly mistakes and improved plan choice satisfaction in a private Medicare Advantage marketplace when an AI tool is used to assist expert agents.

State governments, which regulate Medicaid, marketplace, and nonself-insured employer plans in their state, may represent a viable avenue through which HIL materials can be developed and disseminated to the population. State governments may also create mandates that can support better health insurance decision-making. One such mandate could require all environments in which insurance is sold throughout the state to deploy simulation models with drag-and-drop front-end user interfaces so consumers could understand the total premium and deductible consequences of their plan choices under a range of different utilization scenarios without having to make such calculations themselves. Another option is use of state-based websites to promote HIL education and cognitive burden reduction, such as that hosted by the Colorado Division of Insurance, which provides a “P.R.I.C.E.” tool to educate consumers regarding cost-sharing and price shopping.

Direct consumer assistance is critical but insufficient. Navigators in ACA marketplaces have not been shown to increase overall enrollment but have been demonstrated to increase enrollment among marginalized populations, which may have lower levels of HIL.^8^ Insurance agents and brokers are another form of assistance but are not required to act as fiduciaries.

Effective solutions and pathways are needed; however, the ability to identify them is constrained by the sparse literature on how to best *address* these challenges. The thin evidence base is no doubt a function of poor funding for research to develop HIL interventions. Although federal funding mechanisms such as National Institutes of Health (NIH) are well designed to test existing interventions, they provide limited support for the earlier—yet essential—work of developing those interventions. Designing, piloting and refining effective HIL strategies requires substantial resources, yet this stage remains largely unfunded. Funding exists for test interventions that have already been built and piloted, but funding currently does not exist for the critical pretrial work of *developing* the intervention(s), which itself can be costly and resource intensive. This disconnect creates a critical gap in the research pipeline in generating solutions that are ready for evaluation. Consistent with NIH's shift from funding measurement-focused disparities research to investing in solution-oriented disparities research, a commensurate shift toward solutions is warranted with respect to HIL.

## Supplementary Material

qxag191_Supplementary_Data
